# Interaction Between the Trace Amine-Associated Receptor 1 and the Dopamine D_2_ Receptor Controls Cocaine’s Neurochemical Actions

**DOI:** 10.1038/s41598-017-14472-z

**Published:** 2017-10-24

**Authors:** Aman Asif-Malik, Marius C. Hoener, Juan J. Canales

**Affiliations:** 1 Department of Pharmacology, University of Oxford, Mansfield Rd, Oxford, OX1 3QT United Kingdom; 2 Neuroscience, Ophthalmology and Rare Diseases Discovery & Translational Area, pRED, Roche Innovation Center Basel, F. Hoffmann-La Roche Ltd, Basel, Switzerland; 3 Division of Psychology, School of Medicine, University of Tasmania, Private Bag 30, Hobart, TAS 7001 Australia

## Abstract

Recent evidence suggests that the trace amine-associated receptor 1 (TAAR1) plays a pivotal role in the regulation of dopamine (DA) transmission and cocaine’s actions. However, the underlying mechanisms through which TAAR1 activation mediates these effects have not yet been elucidated. Here, we used fast-scan cyclic voltammetry to measure DA dynamics and explore such mechanisms. We show, first, that the full TAAR1 agonist, RO5256390, dose-dependently blocked cocaine-induced inhibition of DA clearance in slices of the nucleus accumbens. Second, subthreshold inhibition of PKA or PKC phosphorylation did not prevent TAAR1 suppression of cocaine effects whereas subeffective doses of the DA D_2_ receptor antagonist, L-741,626, rescued cocaine’s ability to produce changes in DA uptake in the presence of full TAAR1 activation, thus indicating that TAAR1 modulation of cocaine effects requires simultaneous DA D_2_ receptor activation. Predictably, inhibition of glycogen synthase kinase-3 (GSK-3), which results from activation of D_2_/TAAR1 heterodimers, fully reproduced the inhibitory effects of TAAR1 activation on cocaine-induced changes in DA transmission. Collectively, the present observations reveal that the ability of TAAR1 to regulate cocaine effects is linked to cooperative interactions with D_2_ autoreceptors and associated downstream molecular targets converging on GSK-3 and suggest a new mechanism to disrupt cocaine neurochemical actions.

## Introduction

The trace amine-associated receptor 1 (TAAR1) is a G protein-coupled receptor that is responsive to trace amines (TAs), the major catecholamines and synthetic compounds structurally related to TAs, including amphetamine and its numerous analogues, triggering accumulation of cAMP via adenylyl cyclase activation^1,2^. TAAR1 mRNA and protein expression is enriched in the limbic system and in brain areas associated with the major aminergic pathways, including ascending dopaminergic and serotonergic projections^3–5^. The distribution of TAAR1 is predominantly intracellular, with diffuse expression within the perikaryon and axonal processes and sparse membrane-bound localization at synaptic sites^1,4^, thus being uniquely positioned to regulate aminergic activity. Previous *in vitro* and *in vivo* evidence suggests that TAAR1 stimulation exerts inhibitory control over monoaminergic neurotransmission. Indeed, transgenic mice lacking *Taar1* (*Taar1* ^*−/−*^ mice) exhibited a markedly elevated discharge rate of dopamine (DA) and serotonin (5-HT) neurons in the midbrain^5,6^, and increased DA transmission in the nucleus accumbens (NAc)^7^. Conversely, selective TAAR1 activation with the full agonist, RO5166017, reduced the firing frequency of DA neurons in the midbrain^6^, whereas the selective TAAR1 antagonist, EPPTB, elevated it^8^. This remarkable ability of TAAR1 to regulate DA transmission has spurred a wealth of research into TAAR1 as a target for pharmacological intervention in neuropsychiatry, including addictive disorders^9^.

It is well documented that TAAR1 has the ability to modulate the neurochemical and behavioural effects of psychomotor stimulants. Initial observations showed that the partial agonist, RO5203648, decreased cocaine-stimulated locomotor activity and cocaine self-administration^10^. Partial and full TAAR1 activation similarly prevented the lowering effects of cocaine on brain reward thresholds and the reinforcing and motivational effects of cocaine in a self-administration paradigm^11,12^. Notably, TAAR1 activation blocked cocaine relapse in models of spontaneous renewal, drug-primed and cue-induced reinstatement^12,13^. Although previous *in vitro* research has shown that partial TAAR1 activation reduced cocaine-induced DA overflow in the NAc^12^, the signalling pathways and molecular interactions involved in its modulation of cocaine-induced changes in DA uptake, which underlie the reinforcing and euphoric effects of cocaine^14,15^, are unknown. Delineating such pathways is crucial to develop and optimize TAAR1-based treatments for addiction and other disorders associated with DA dysfunction.

TAAR1’s cellular distribution allows this receptor to regulate aminergic transmission by way of interactions with transporter sites, presynaptic autoreceptors and associated intracellular signalling cascades^9^. TAAR1 stimulation triggers accumulation of cAMP via Gα_s_-adenylyl cyclase activation which can, in turn, promote PKA and PKC phosphorylation^1–3,16^, and also activates a G protein-independent, β-arrestin2-dependent pathway involving protein kinase B (AKT)/glycogen synthase kinase-3 (GSK-3)^17^, which is modulated by DA D_2_ receptors^18^. Although such widespread molecular interactions complicate the identification of the mechanisms responsible for TAAR1’s capacity to regulate cocaine’s neurochemical actions, here we used *in vitro* fast-scan cyclic voltammetry to monitor changes in electrically evoked DA transmission produced by cocaine and aimed to characterize the underlying substrates linked to TAAR1’s ability to regulate the neurochemical actions of cocaine.

## Methods

### Tissue preparation

**Figure 1:**
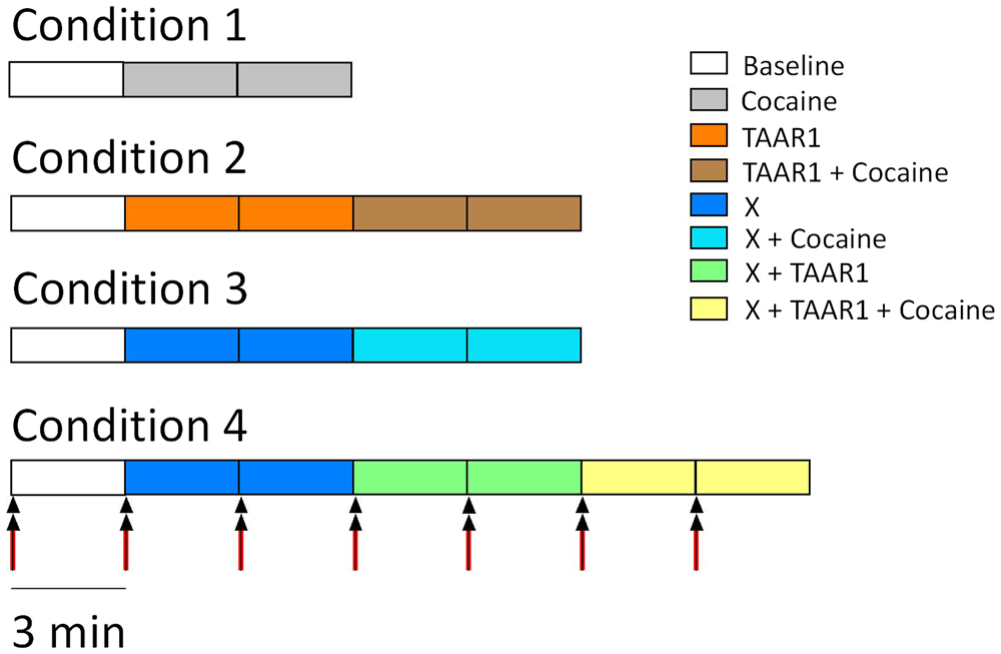
Diagrammatic representation of the protocol used for each experimental drug condition. A 350 μA stimulation was passed every 3 m (red arrows), at 5 s passed the start of the 3 min file (set at 50 Hz with 10 pulses and a 1 ms pulse width), which is presented as each coloured rectangle. Drugs were perfused 3 min before the following stimulation so as to observe drug effects during the corresponding stimulation. Two electrically-stimulated responses were used to calculate an average effect for each condition. X corresponds to any of the agonists/antagonists used in these experiments.

Brain slices from 58 male Lister Hooded rats were used for this study. The experiments were carried out under institutional ethics approval (AWERB Sub-committee, University of Leicester) and appropriate project and personal license authority granted by the UK Home Office under the Animals (Scientific Procedures) Act 1986. Prior to use, animals were housed on a 12 h light/dark cycle with *ad libitum* access to food and water. On the day of the experiment, a rat was anaesthetized with isofluorane and culled via a schedule 1 procedure (under the Animals Scientific Procedures Act 1986, Amendment Regulations 2012). The brain was rapidly removed and placed in a tube containing pre-carboxygenated (i.e. bubbled with 95% O_2_ and 5% CO_2_), ice-cold, sodium-free slicing artificial cerebrospinal fluid (S.aCSF), so as to prevent synaptic transmission during slicing, consisting of 250 mM sucrose (Merck Group, Germany), 2.5 mM KCl (Sigma-Aldrich, UK), 11 mM d-glucose (Sigma-Aldrich, UK), 1.2 mM NaH_2_PO_4_ (Sigma-Aldrich, UK), 25 mM NaHCO_3_ (Sigma-Aldrich, UK), 0.4 mM l-ascorbic acid (Sigma-Aldrich, UK), 0.1 mM CaCl_2_ (Sigma-Aldrich, UK), and 4 mM MgCl_2_ (Thermo Fisher Scientific, Belgium), and adjusted to pH 7.4. The brain was then sectioned in ice-cold carboxygenated S.aCSF on a Vibratome 1000 Classic vibrating microtome (The Vibratome Company, MO, USA). Coronal slices (400 µM) of the striatum containing the NAc were maintained at room temperature in continuously carboxygenated experimental aCSF (E.aCSF), which consisted of 126 mM NaCl, 2.5 mM KCl, 11 mM d-glucose, 1.2 mM NaH_2_PO_4_, 25 mM NaHCO_3_, 0.4 mM l-ascorbic acid, 2.4 mM CaCl_2_, 1.2 mM MgCl_2_ and adjusted to pH 7.4. Slices were allowed to recover for at least 30 min at room temperature before use. The various experimental manipulations started 5–10 min after transfer to the FSCV slice chamber (see below and Fig. 1) to allow slices to equilibrate in warmed aCSF.

### FSCV electrodes

Recording electrodes were manufactured as described previously^19^. A single carbon fiber (7 µm in diameter; Goodfellow, Cambridge Ltd.) was aspirated into a borosilicate glass capillary (100 mm length, 1/0.58 mm OD/ID; World Precision Instruments, FL, USA). The capillary was then pulled to a fine tip using a vertical needle puller (PE-21, Narishige, Japan) and the exposed carbon fibre was cut, using a scalpel, to 100 µm in length. A silver conductive paint (Coating Silver Print II, GC Electronics, USA) coated piece of wire was inserted into the capillary, secured with a gold pin (Newark, IL, USA) and heat shrink-wrapped to the capillary (FP-301, 3 M). Each electrode was tested to ensure a suitable background (non-Faradaic) current profile by applying the voltage waveform at 60 Hz. Each good electrode was then cycled at 60 Hz for a maximum of 30 min from −0.4 to 1.3 V and back (versus an Ag/AgCl reference) at a ramp of 400 V/s and application frequency of 60 Hz until stable. Stability was determined when the baseline recording showed a drift of no more than 2 nA over 30 s. Once stable, electrodes were cycled from −0.4 to 1.3 V and back at 400 V/s at a frequency of 10 Hz until the drift was again minimal. Reference electrodes were manufactured with a piece of silver wire coated in KCl (Ag/AgCl) and attached to a silver pin (Newark, IL, USA). Bipolar stimulating electrodes were purchased directly (FHC, ME, USA).

### FSCV equipment

The FSCV setup was custom built, consisting of a slice chamber, stimulating, recording and reference electrodes connected to a computer and amplifier. The recording and reference electrodes were connected to a potentiostat and head stage circuit (ChemClamp, Dagan Instruments USA) and a computer running Demon Voltammetry Software (Wakeforest Innovations, NC, USA). Two data acquisition cards (NI-DAQ; PCI-6711 and PCI-6052e; National Instruments, Austin, TX) were used for interfacing Demon Voltammetry with a Chem-Clamp potentiostat (Dagan Corporation; Minneapolis, MN) for voltammetric recordings. The NI-DAQ cards contain multiple on-board high-speed clocks, and several 16 bit analogue outputs/inputs suitable for generating potential sweeps and acquiring voltammograms at high rates (>100 kHz) while performing electrical stimulations for evoking DA release. NI-DAQ cards were connected to the potentiostat via specialized breakout boxes created locally from 2 NI-DAQ CB-68LPR screw terminals^20^. The recording electrode potential was linearly scanned at a rate of 400 V/s as a triangular waveform from −0.4 V to 1.3 V and back to −0.4 V vs the reference electrode. Cyclic voltammograms were recorded at the recording electrode every 100 ms by means of the voltammeter (Dagan Instruments, USA). At this waveform, DA oxidizes at ~ 0.6 V and reduces at ~−0.2 V.

### FSCV recordings

**Figure 2:**
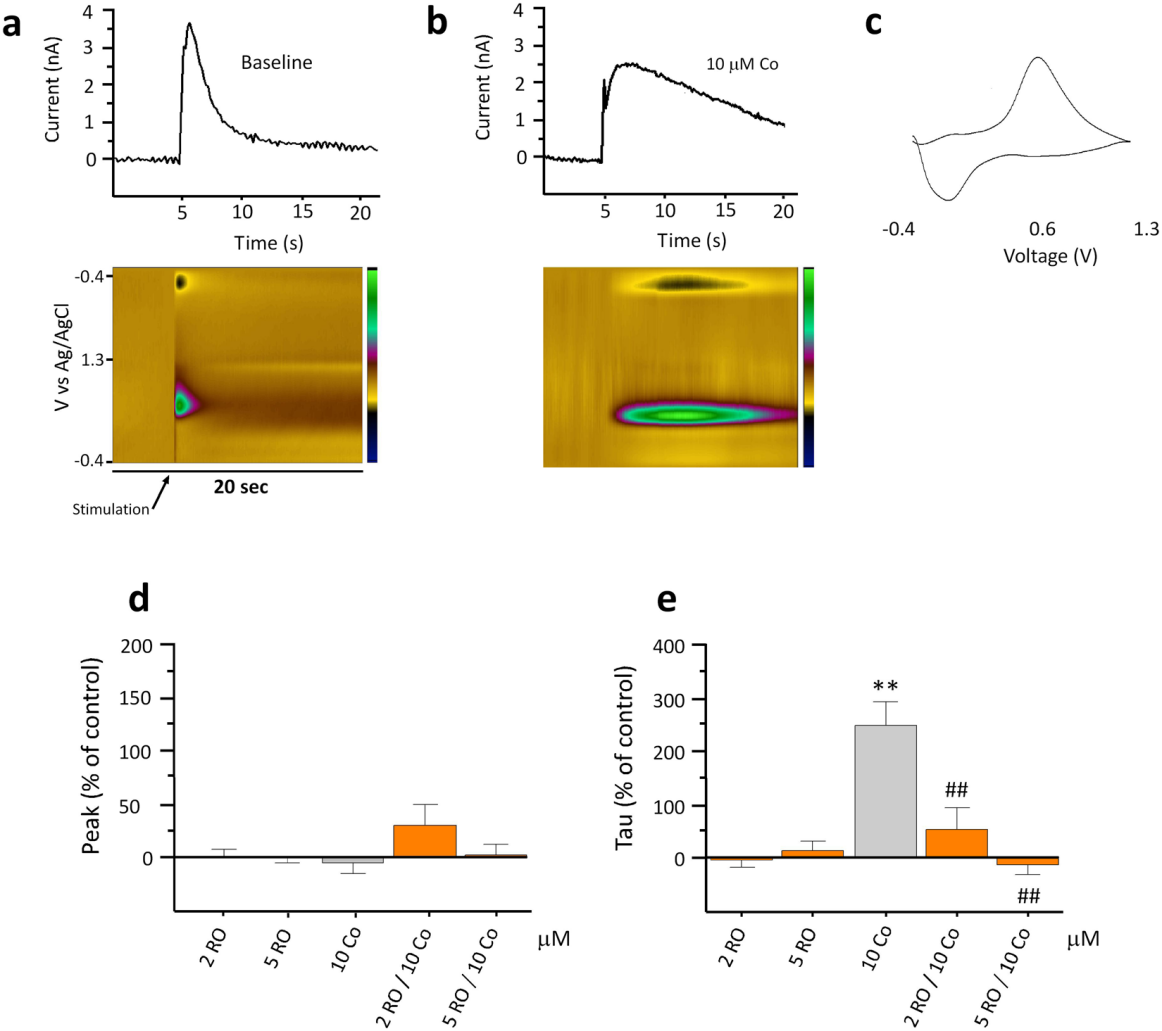
RO5256390 reduces cocaine-induced increases in DA transmission in slices of rat NAc. Representative traces demonstrate the effect of cocaine (Co; 10 μM) perfusion on electrically-stimulated (at 350 μA; 10 pulses set at 50 Hz with a 1 ms pulse width) DA outflow in comparison to baseline in raw current values (nA) (**a**). The colour plots represent the voltammetric currents (encoded in colour in the z-axis) plotted against time (x-axis) (**b**). An example of the background-subtracted cyclic voltammogram, which identified the detected analyte as DA (**c**). Application of 10 μM Co resulted in a significant decrease in DA uptake which was significantly attenuated by both 2 μM and 5 μM RO5256390 but had no effect on DA release (**d,e**). (*p < 0.05, **p < 0.01 vs baseline; ^#^p < 0.05, ^##^p < 0.01 vs cocaine values; n = 7–19).

For recordings, a slice was placed in the FSCV slice chamber, held in place with a purpose-built grid and superfused with continuously carboxygenated E.aCSF at a flow rate of 1.4 ml/m heated with a purpose-built peltier to 32–33°C. Waste E.aCSF was aspirated at the same flow rate from the other end of the slice chamber. Flow and aspiration rate was controlled with a Minipulse peristaltic pump (Gilson, Bedfordshire, UK). The recording electrode was positioned ~75 µm below the surface of the slice in the NAc. DA release was electrically evoked at 50 Hz every 3 min by a 1 ms, ten-pulse stimulation (monophasic, 300 µA) using the stimulating electrode placed 100–200 µm from the recording electrode within the NAc. Current pulses were generated by the acquisition software and applied via an ISo-Flex stimulus isolator (A.M.P. Instruments, Jerusalem, Israel). Voltage waveforms (10 Hz) were applied to electrodes using Demon software and the resulting changes to current were recorded and analysed. The recording electrode generates a characteristic background signal that was subtracted to yield the Faradaic current caused by oxidation and reduction of DA. DA was confirmed in each recording by observation of the cyclic voltammogram (noting the position of oxidation and reduction peaks; for e.g. Fig. 2c) and colour plots permitted the visualization of release dynamics over time (Fig. 2a,b).

Drugs were applied by superfusion at the same time as the recording was initiated. Slices were randomly assigned to the different trials. Trials consisted of 9, 15 or 21 min runs with an electrical stimulation administered every 3 min of either no drug, 1 or 10 µM cocaine (Sigma, UK), 2 or 5 µM of the full TAAR1 agonist, RO5256390 (Hoffmann-La Roche Ltd., Switzerland), followed by 6 min of co-application of RO5256390 (2 or 5 µM) with 10 µM cocaine (see Fig. 1). The effects of cocaine on DA outflow was found to return to a stable pre-stimulation level within 3 min and concentrations of all other drugs used in this study were chosen on the basis of no effect on DA transmission when perfused alone. This enabled us to take two reliable peak and tau measures for each condition within each slice and calculate the average prior to gathering the total average for each condition from all slices.

The lower concentration of cocaine tested in this experiment (1 μM) produced small increases in release but no changes in DA clearance (results not shown) and the higher cocaine concentration demonstrated consistently robust effects on DA overflow. Thus, we decided to use the higher concentration of cocaine throughout the rest of the study to investigate the mechanisms through which TAAR1 modulates cocaine-induced alterations in DA uptake.

The following drugs were also used: phorbol 12-myristate 13-acetate (PMA) [protein kinase C (PKC) activator; 0.1 or 0.3 µM;^21,22^] (Sigma-Aldrich, UK), Go 6983 (PKC inhibitor; 0.1, 2 or 10 µM^21^; (Sigma-Aldrich, UK), 3′, 5′- cyclic adenosine monophosphate sodium salt (cAMP) [protein kinase A (PKA) activator; 3 or 10 µM^23^] (Santa Cruz Biotechnology, TX, USA), KT 5720 [PKA inhibitor; 2 nM, 20 nM or 0.1 µM^24,25^] (Sigma-Aldrich, UK), sumanirole (D_2_ receptor agonist; 0.1 or 0.3 µM^26^; Sigma-Aldrich, UK), L-741,626 (D_2_ receptor antagonist; 3 nM, 30 nM or 0.1 µM^26^; Santa Cruz Biotechnology, CA, USA) and SB216763 [GSK-3 inhibitor; 0.1 µM or 1 µM^27^; (Cell guidance systems, Cambridge, UK)]. Concentrations for all compounds were selected on the basis of empirical data involving neurochemical or electrophysiological actions in slice preparations (as referenced above) and adjusted based on their effects in our own preparation, but always to lower doses than those previously reported. Only concentrations of drugs that had an effect on cocaine-induced DA transmission but no significant effect on DA clearance in their own right were used for ease of interpretation. Each slice was perfused with only one drug in the absence and presence of cocaine. In each condition, two electrically stimulated responses, over the time course of six minutes were taken from each slice. In order to ensure there was no effect on either peak or tau measures from cumulative stimulation within the entire length of the longest experiment (21 min), slices were stimulated every 3 min in the absence of any drug and no effect on either peak or tau between the first and last stimulation was confirmed, thus enabling us to confidently base our findings on drug treatment.

Background subtracted cyclic voltammograms were obtained by subtracting the current obtained at the point before stimulation of every experiment, before drugs were superfused into the slice chamber. The peak oxidation current (nA) for DA in each voltammogram has been deemed an appropriate measure of DA release and tau an adequate measure of DA reuptake. Tau is a measure of exponential decay from peak to baseline^20^. The *n* value represents number of slices.

### Statistical Analysis

All assessments are reported in relation to percent from baseline. Recorded current (nA) vs time data were extracted from Demon Voltammetry Software and exported to a tab delimited file, compatible with Microsoft Excel. Percentage increases or decreases from baseline of DA release (i.e. peak) and DA reuptake (i.e. tau) in the presence of any treatment was analysed. Data were analysed by one-way ANOVA to compare the effects of the various manipulations across groups followed by *post-hoc* Fisher’s Least Significant Test comparisons. Additionally, we conducted a t-test to compare the effects of specific drugs against their own baseline where applicable. Statistical significance was set at α = 0.05 for all experiments. All statistical analyses were performed using StatView 5.0 (SAS Institute, NC, USA).

## Results

### Effects of cocaine on DA transmission

Cocaine (10 μM) was superfused onto NAc brain slices for a total of 9 min. Stimulation was applied at the beginning of every third min allowing a measure of basal DA transmission (i.e. the electrically stimulated response in the absence of any drug) and two measures of cocaine-induced changes in DA transmission. The average effects (peak and tau) of two cocaine-induced changes in DA transmission were compared to the basal values, and percentage increases/decreases of either peak or tau in the presence of a drug were analysed. The background-subtracted cyclic voltammogram identified the detected analyte as DA (Fig. 2c) and colour plots represented the voltammetric currents (encoded in colour in the z-axis) plotted against the applied potential (y-axis) and time (x-axis) (Fig. 2b,c).

DA peak response was unaffected by cocaine in comparison to the average baseline peak response (Fig. 2d; −4.42 ± 10.09%, n = 19), whereas tau was significantly increased in the presence of 10 μM cocaine (Fig. 2e; 258.50 ± 44.20%; F_(1,18)_ = 9.27, p = 0.007, n = 19).

### Effects of TAAR1 activation on DA transmission

To investigate whether TAAR1 activation affected DA transmission when applied alone, NAc slices were superfused with two concentrations of RO5256390 (2 μM or 5 μM). The average of two stimulus-induced responses was calculated and percentage deviation from baseline (i.e. stimulus-induced DA transmission in the presence of no drug) responses in each slice was analysed by *t*-test. Both the low (mean peak 0.40 ± 11.28%, mean tau −4.35 ± 12.35% deviation from baseline) and the high (−0.11 ± 4.20%, mean tau 15.31 ± 16.84% concentrations of RO5256390 were without effect by themselves on either peak DA or tau (Fig. 2d,e; n = 10–13).

### TAAR1 modulation of cocaine-induced inhibition of DA clearance

To investigate TAAR1’s effect on cocaine-stimulated DA overflow, brain slices were perfused with cocaine (10 μM) and RO5256390 (2 μM or 5 μM) in combination. The mean peak and tau deviations from baseline for 10 μM cocaine in the presence of 2 μM RO5256390 were 36.93 ± 26.07% and 52.86 ± 43.04% respectively; and those for 10 μM cocaine in the presence of 5 μM RO5256390 were 3.36 ± 9.48% and −13.59 ± 17.24% respectively. ANOVA revealed a significant main effect of treatment for RO5256390 (Fig. 2d,e; F_(4,52)_ = 11.76, p < 0.001, n = 15) and post-hoc analysis showed that cocaine-induced changes in tau were dose-dependently attenuated by the full TAAR1 agonist (p < 0.01).

### Effects of selective PKA activators and inhibitors on cocaine-induced changes in DA transmission

**Figure 3:**
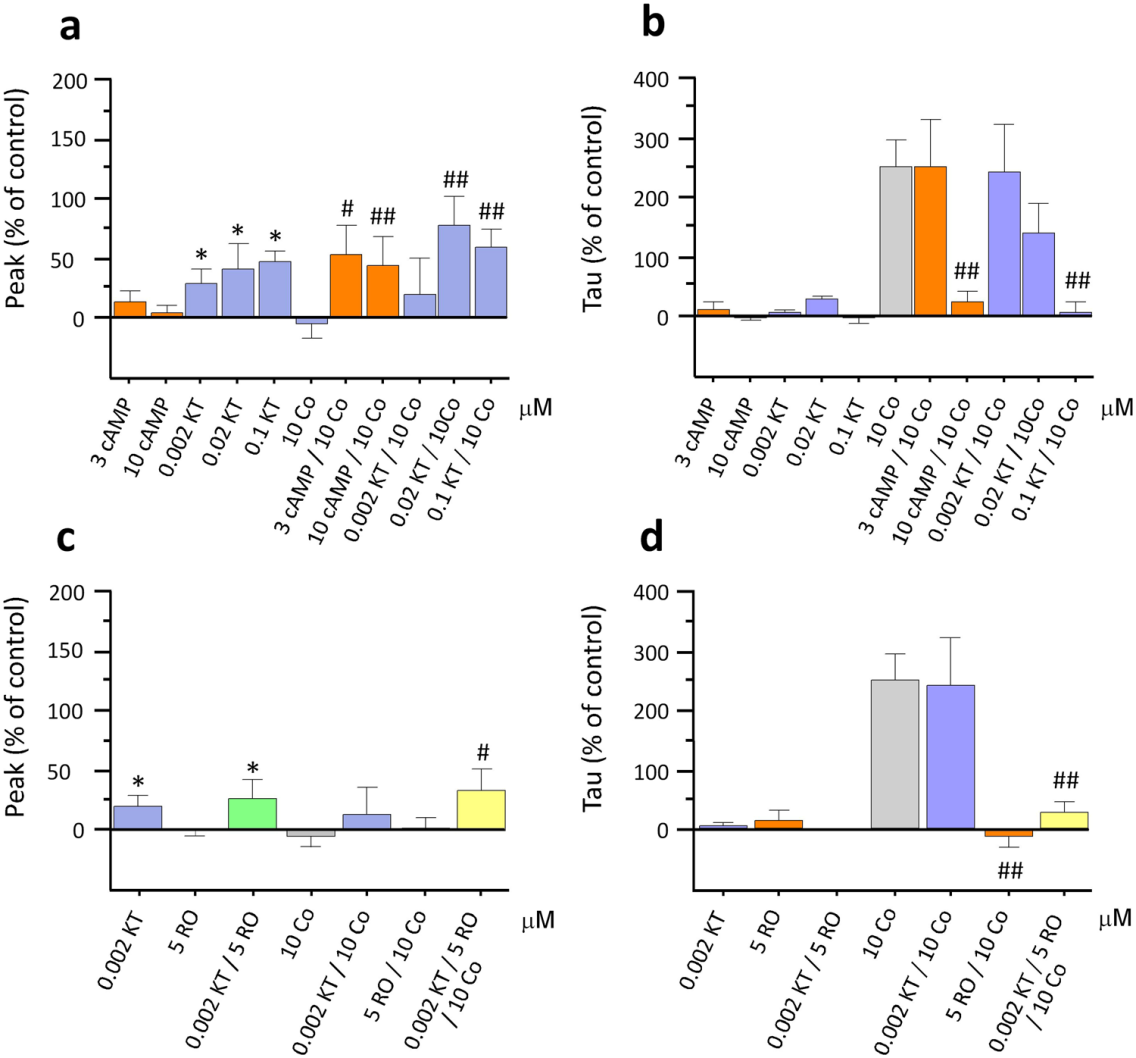
Effects of PKA inhibition and activation on cocaine-induced changes in DA transmission. Co-application of 10 μM cocaine (Co) with either a PKA activator (cAMP) or an inhibitor (KT 5720; KT) resulted in a significant increase in DA release (**a**). KT also caused a significant increase in peak DA in comparison to baseline values when perfused alone (**a**). Superfusing cocaine with either 10 μM cAMP or 0.1 μM KT significantly attenuated cocaine-induced increases in tau (**b**). Perfusion of the lowest concentration of KT, which had no effect on DA clearance in its own right, in the presence of both 5 μM RO5256390 and 10 μM cocaine, had no effect on RO5256390’s ability to attenuate cocaine-induced increases in tau (**d**). (*p < 0.05, **p < 0.01 vs baseline; ^#^p < 0.05, ^##^p < 0.01 vs cocaine values; n = 5–19).

We targeted individual pathways that are known to be involved in TAAR1’s downstream signalling cascades^9^ to assess their impact on cocaine’s effects and their influence on TAAR1’s modulation of cocaine’s effects. The PKA activator (cAMP) and the inhibitor (KT 5720) were superfused alone and in the presence of cocaine so as to ensure effects observed were due to the activator and inhibitor in their own right. All concentrations of KT 5720 were found to significantly increase DA release in comparison to baseline (Fig. 3a; 36.34 ± 16.67% peak deviation, 28.25 ± 5.27% tau deviation; F_(1,4)_ = 15.08, p = 0.018, n = 5; 20 nM and 39.39 ± 9.69% peak deviation, −1.45 ± 10.69% tau deviation F_(1,4)_ = 7.39, p = 0.042, n = 5; 100 nM; see below for 2 nM KT 5720). In addition, by activating or inhibiting the PKA pathway, we significantly altered cocaine-induced changes in DA transmission (Fig. 3a; F_(10,77)_ = 2.28, p = 0.021, n = 29). Both cAMP (45.42 ± 23.08% and 36.76 ± 22.08% for 3 μM cAMP and 10 μM cAMP respectively) and KT 5720 (68.36 ± 19.64% and 50.27 ± 13.64% for 20 nM and 100 nM KT 5720 respectively) significantly potentiated the effect of cocaine on peak DA. Post-hoc analysis showed that this effect was greater in the presence of the lower concentrations of both the activator and inhibitor (3 μM cAMP and 20 nM KT 5720; p < 0.01). In addition, both the activator and the inhibitor dose-dependently attenuated cocaine’s effect on tau (Fig. 3b; F_(10,77)_ = 7.11, p < 0.001, n = 29). The mean tau deviations from baseline for 10 μM cocaine in the presence of 3 and 10 μM cAMP was 248.01 ± 82.25% and 21.87 ± 21.16% respectively; and those for 10 μM cocaine in the presence of 20 and 100 nM KT 5720 were 137.76 ± 50.01 and 7.29 ± 15.13% respectively.

In order to establish whether the observed effects of inhibiting the PKA pathway played a role in TAAR1’s ability to regulate cocaine-induced changes in DA transmission, we superfused KT 5720 (2 nM) together with both RO5256390 and cocaine. This concentration of KT 5720 was established as ineffective on cocaine’s ability to increase tau (241.43 ± 79.83% deviation from baseline) but was found to significantly increase DA release when perfused alone in comparison with baseline responses (Fig. 3a; 25.34 ± 10.49%; F_(1,12)_ = 6.47, p = 0.026, n = 13). Therefore, in order to ascertain whether or not PKA played a role in TAAR1’s ability to alter cocaine effect on DA transport, we used this low concentration of KT 5720. We found that in the presence of 2 nM KT 5720, TAAR1’s ability to modulate cocaine’s effect on DA reuptake was unaffected (Fig. 3d; 28.66 ± 16.73%; F_(6,65)_ = 10.93, p < 0.001, n = 7). In addition, perfusion of both 2 nM KT 5720 and 5 μM RO5256390 had no effect on tau in the absence of cocaine (2.80 ± 7.26% deviation from baseline), although they did induce a similar significant increase in peak as observed with 2 nM KT 5720 alone (32.32 + 19.33% deviation from baseline).

### Effects of selective PKC activators and inhibitors on cocaine-induced changes in DA transmission

**Figure 4:**
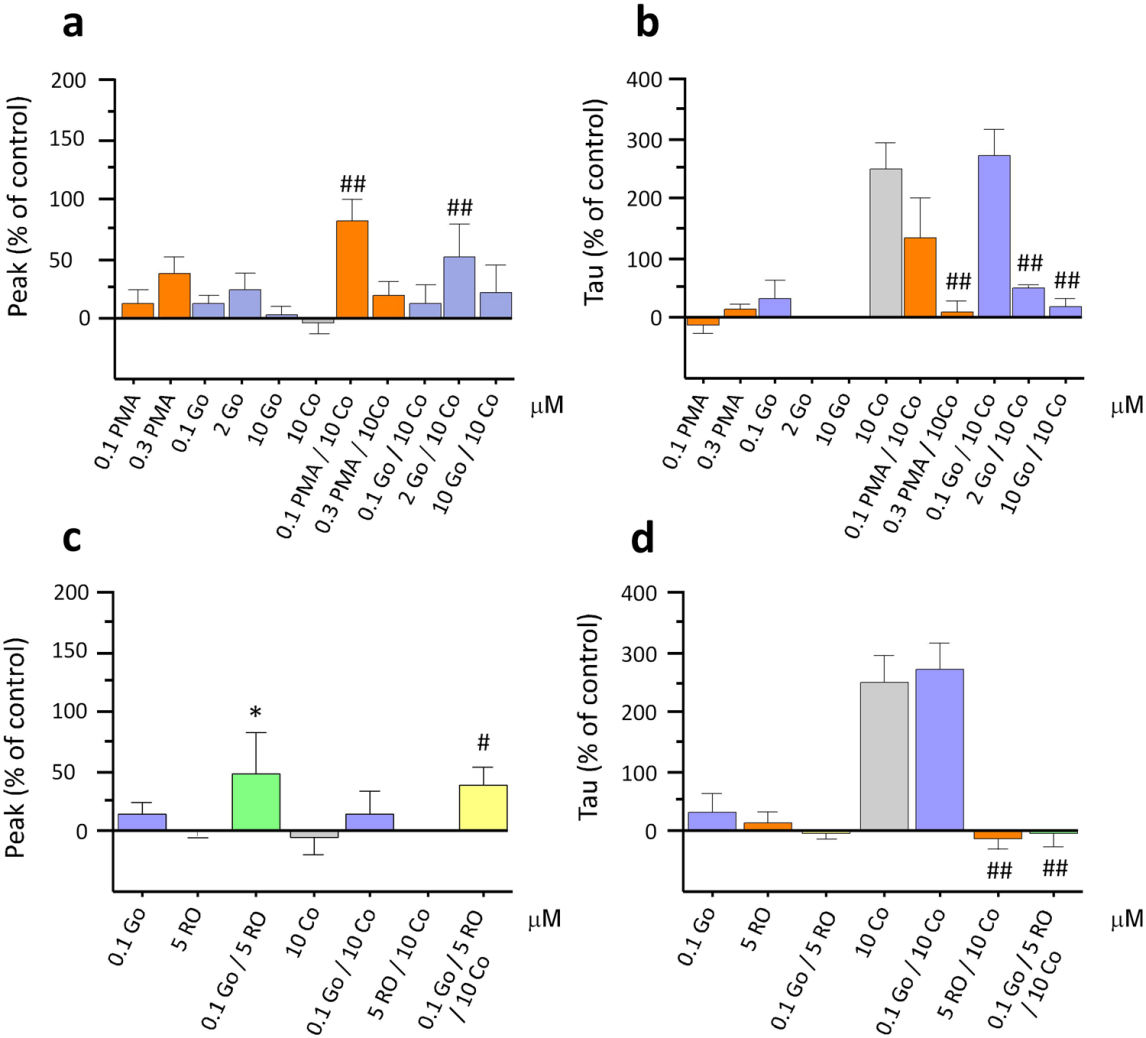
Effects of PKC inhibition and activation on cocaine-induced changes in DA transmission. Co-application of the lower concentrations of both PKC activator (PMA) and PKC inhibitor (Go 6983; Go) with cocaine (Co) resulted in a significant increase in peak DA (**a**). Both PMA and Go dose-dependently increased DA clearance in the presence of Co (**b**). Although 100 nM Go was without effect, there was a significant increase in DA peak compared to baseline values when perfused in combination with RO5256390. In the presence of 100 nM Go, RO5256390 still produced an attenuation of cocaine’s effect on tau (*p < 0.05, **p < 0.01 vs baseline; ^#^p < 0.05, ^##^p < 0.01 vs cocaine values; n = 7–19).

We then aimed to establish any possible effects of modulating the PKC pathway in a similar fashion by superfusing an activator (PMA) and an inhibitor (Go 6983) of PKC, alone and in the presence of cocaine. The selective PKC activator PMA was superfused at two different concentrations, 100 nM (15.09 ± 11.20% deviation from baseline peak and −12.60 ± 11.68% deviation from baseline tau) and 300 nM (42.87 ± 16.58% deviation from baseline peak and 14.03 ± 9.73% deviation from baseline tau) and the inhibitor Go 6983 was superfused at 100 nM (13.74 ± 8.99% deviation from baseline peak and 32.48 ± 30.52% deviation from baseline tau), 2 μM (27.36 ± 16.03% deviation from baseline peak and 2.91 ± 5.00% deviation from baseline tau) and 10 μM (2.35 ± 10.12% deviation from baseline peak and 2.75 ± 11.64% deviation from baseline tau). A significant effect of treatment was found on both cocaine-induced peak DA (Fig. 4a; F_(10,73)_ = 2.73, p = 0.007, n = 29) and tau (Fig. 4b; F_(10,73)_ = 7.53, p < 0.001, n = 29) and post-hoc analyses revealed a significant increase in cocaine-induced peak DA following perfusion of 2 μM Go 6983 (58.48 ± 31.60%) and 100 nM PMA (92.70 ± 21.86%) (p < 0.01). Further, a dose-dependent reduction of tau on co-application of cocaine with both the activator and the inhibitor was observed (p < 0.01). The mean tau deviations from baseline for 10 μM cocaine in the presence of 100 nM and 300 nM PMA was 136.60 ± 66.12% and 8.52 ± 18.51% respectively; and those for 10 μM cocaine in the presence of 100 nM, 2 μM and 10 μM Go 6983 were 270.52 ± 44.62%, 48.57 ± 7.92 and 18.47 ± 13.91% respectively.

We then superfused the selective PKC inhibitor Go 6983 at the inert concentration of 0.1 μM with RO5256390 and cocaine to establish whether the PKC pathway played a role in TAAR1’s ability to modulate cocaine-induced DA changes in uptake. In the presence of Go 6983, RO5256390 was still able to completely block cocaine’s effect on tau (Fig. 4d; −0.59 ± 25.02%; F_(6,63)_ = 11.01, p < 0.001, n = 6) and thus the PKC inhibitor did not affect TAAR1’s ability to modulate cocaine-induced changes in DA clearance. A significant increase in DA peak did occur, however, on co-application of this inert concentration of Go 6983 and RO5256390 (Fig. 4c; 45.20 ± 33.82%; F_(1,4)_ = 10.989, p = 0.030, n = 5), indicating that although this concentration of the PKC inhibitor had no effect on cocaine-induced increases in tau, it was physiologically active.

### Modulation of DA clearance by TAAR1 is inhibited by DA D2 receptor antagonism

**Figure 5:**
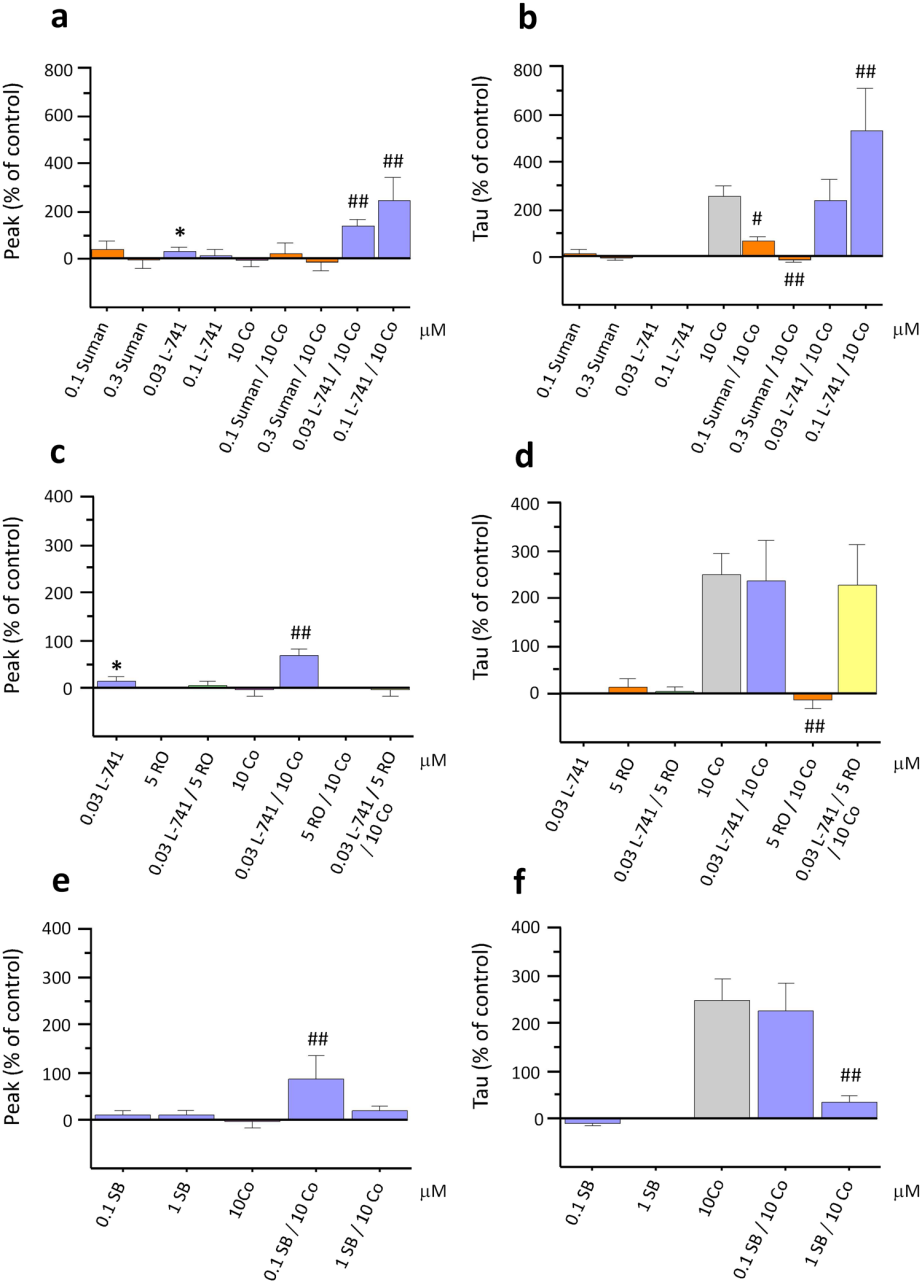
The DA D_2_ receptor antagonist, L-741,626, blocks RO5256390’s effects on cocaine-induced changes in DA transport. Co-application of cocaine (Co) with the DA D_2_ receptor antagonist, L-741,626 (L-741), dose-dependently increased cocaine effects on DA release (**a**). This effect was also observed with the perfusion of the lower concentration of L-741 alone. Sumanirole (Suman), DA D_2_ receptor agonist dose-dependently produced a significant attenuation of cocaine’s effect on tau whereas L-741,626 significantly potentiated cocaine’s effect on tau (**b**). The lower concentration of L-741,626 (30 nM), which did not affect cocaine-induced changes in DA clearance when perfused with RO5256390 and cocaine, completely blocked RO5256390’s ability to inhibit cocaine’s effects on DA uptake (**d**). SB216763 (SB), a GSK-3 inhibitor, increased peak at the lower concentration (**e**) and significantly attenuated cocaine-induced DA increases in tau at the higher concentration (**f**). (*p < 0.05, **p < 0.01 vs baseline; ^#^p < 0.05, ^##^p < 0.01 vs cocaine values; n = 5–19).

To investigate whether D_2_ receptors play a significant role in TAAR1’s ability to modulate cocaine-induced DA overflow, we perfused the highly selective and potent D_2_ receptor agonist, sumanirole, at two different concentrations (0.1 and 0.3 μM) both alone at 100 nM (19.90 ± 18.82% deviation from baseline peak and 8.56 ± 18.43% deviation from baseline tau) and 300 nM (−3.05 ± 16.31% deviation from baseline peak and −0.92 ± 10.77% deviation from baseline tau) and in the presence of cocaine. Sumanirole, with an EC_50_ between 17–75 nM^28^, has more than 200-fold selectivity for the D_2_ receptor subtype in comparison to other DA receptor subtypes. ANOVA revealed a significant effect of treatment on cocaine-induced increases in tau (Fig. 5b; F_(8,67)_ = 7.89, p < 0.001, n = 10). Sumanirole exposure produced a significant, dose-dependent reduction in tau on co-application with cocaine (61.92 ± 25.54% and −17.09 ± 6.08% at the lower and higher concentration respectively; p < 0.01). No effect of sumanirole treatment was found on cocaine’s effect on peak DA (Fig. 5a; 10.88 ± 23.94% and −7.32 ± 16.59% with 100 nM and 300 nM respectively; n = 10).

We then sought to further assess the role of D_2_ receptors on cocaine-induced increases in tau by using the selective D_2_ receptor antagonist, L-741,626. A significant, dose-dependent increase in DA release on co-application of cocaine with both concentrations of L-741,626 was observed (68.34 ± 13.31% and 235.17 ± 84.38% respectively), as shown by ANOVA and post-hoc analysis (Fig. 5a; F_(8,67)_ = 4.63, p < 0.01, n = 14). In addition, post-hoc analyses also showed that at the higher concentration, L-741,626 significantly increased cocaine-induced changes in tau (Fig. 5b; 523.33 ± 176.67%; p < 0.01).

We went on to evaluate the effects of the D_2_ receptor antagonist, L-741,626, on TAAR1’s ability to modulate DA transmission by superfusing 10 μM cocaine with both 30 nM L-741,626 and 5 μM RO5256390. We selected the 30 nM of L-741,626 because the previous experiment indicated that this dose did not affect tau following single application or in combination with cocaine, although it still was able to produce changes in DA release in comparison to basal values (Fig. 5c; 122.61 ± 48.66%; F_(1,15)_ = 8.461, p < 0.011, n = 16). A similar increase in tau on co-application of L-741,626 with RO5256390 and cocaine, in comparison to cocaine alone, was observed (Fig. 5d; 288.47 ± 84.06%; F_(6,69)_ = 9.754, p < 0.001, n = 7). Therefore, at concentrations that were devoid of effects on tau following single application, L-741,626 blocked RO5256390’s ability to inhibit cocaine-induced increases in tau and thus the ability of TAAR1 to modulate cocaine’s effects on DA clearance. Interestingly, the opposite was observed in peak, where RO5256390 inhibited the peak increase induced by L-741,626 (Fig. 5c,d; 6.24 ± 8.21%).

### Cocaine-induced increases in tau are blocked by GSK-3 inhibition

In order to further confirm whether the TAAR1/D_2_R interactions are crucial for TAAR1 to regulate cocaine-induced alterations in DA transmission, we inhibited the GSK-3 pathway using the selective GSK-3 inhibitor, SB216763. Since GSK-3 is activated by D_2_ alone via the β-arrestin2/AKT pathway, whereas the heterodimer TAAR1/D_2_ inhibits GSK-3 via the same pathway, thus by inhibiting GSK-3, we expected GSK-3 inhibition to mimic TAAR1 activation effects. SB216763 was superfused at two different concentrations (0.1 and 1 μM), both alone and in the presence of cocaine. No effect was observed of either concentration of SB216763 on DA transmission when perfused alone (10.14 ± 9.59%, 10.12 ± 7.90% deviation from baseline peak and −5.59 ± 6.10%, 0.75 ± 3.25% deviation from baseline tau). ANOVA showed an effect of treatment on both cocaine-induced changes in peak DA (Fig. 5e; F_(4,40)_ = 3.14, p = 0.024, n = 13) and tau (Fig. 5f; F_(4,40)_ = 7.43, p = 0.001, n = 13). Post-hoc analyses showed that the lower concentration of SB216763 caused a significant increase in cocaine-induced DA release (87.90 ± 48.400; p < 0.01) and that cocaine effects on tau were dose-dependently attenuated by SB216763 (226.774 + 58.921 and 35.199 + 16.004 respectively; p < 0.01).

## Discussion

**Figure 6:**
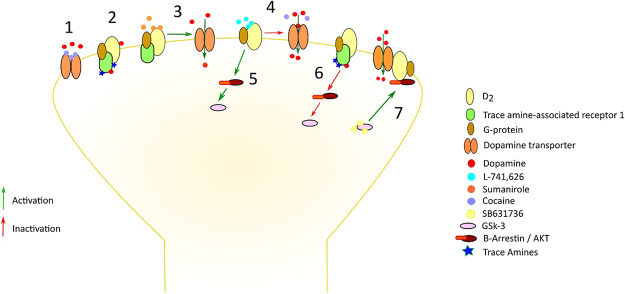
Proposed mechanism of TAAR1’s role in modulating dopamine (DA) transmission fluctuations induced by cocaine. Cocaine blocks dopamine transporter (DAT) function and thus inhibits DA reuptake by binding to the DAT (1). The DA D_2_ receptor is a G-protein-coupled receptor, which, when attached to TAAR1 can form a heteromeric complex^52^. This complex potentiates DA D_2_ receptor-mediated pre-synaptic autoinhibition and inhibits DA D_2_ receptor-mediated post-synaptic signalling. As a sentinel system, TAAR1 is sensitive to shifts in DA concentrations and promotes DA homeostasis (2). Sumanirole, a DA D_2_ receptor agonist causes a similar inhibition of cocaine-induced changes in DA uptake (3) and the antagonist L-741,626 potentiates cocaine-induced effects on DA clearance (4). DA D_2_ receptor stimulation activates GSK-3 β-arrestin2-dependent pathway (5) and the TAAR1/DA D_2_ receptor heteromeric complex inhibits GSK-3 through the same pathway (6). Thus, inhibiting GSK-3 with SB631736 activates AKT, which is bound to D_2_/DAT complex^53^, increasing DA reuptake (7).

This study set out to examine the ability of TAAR1 to regulate the neurochemical effects of cocaine, measuring DA release and uptake dynamics in the NAc in real time. The results provided a straightforward demonstration that TAAR1 activation completely prevented the effects of cocaine on DA uptake, which we showed to require co-activation of DA D_2_ autoreceptors, but not the recruitment of its associated PKA and PKC signalling cascades. Moreover, the data revealed that such cooperative interactions between TAAR1 and DA D_2_ receptors are likely to occur through inhibition of the β-arrestin2-dependent pathway, since GSK-3 inhibition fully reproduced the effects of TAAR1 activation on cocaine-induced changes on DA clearance, though it increased DA release on co-application of cocaine. These findings uncover the primary mechanisms through which TAAR1 is able to regulate the alterations in DA transmission produced by cocaine (Fig. 6) and underscore the potential of TAAR1 as a target for stimulant addiction treatment.

Cocaine is a psychomotor stimulant that exhibits rapid brain uptake and relatively short half-life (ca. 20 min), acting primarily by blocking the DAT, preventing DA reuptake and producing subsequent elevations of extracellular levels of DA^29^. The characteristic pharmacokinetic profile of cocaine and the increases it produces in extrasynaptic DA are believed to mediate its reinforcing and euphoric effects^30,31^. In addition, enhanced mesolimbic DA transmission is triggered by exposure to cocaine-related stimuli^32^ and coincides with the initiation of cocaine seeking behaviour^33^. Considerable experimental evidence indicates that DA transmission is strongly modulated by TAAR1. In patch clamp preparations, the full TAAR1 agonist, RO5256390, attenuated DA neuron firing in the ventral tegmental area^6^, whereas the partial agonist, RO5263397, augmented it under conditions of low neuronal discharge, as did the antagonist EPPTB^8^. These data suggests that TAAR1 is constitutively active and/or tonically activated by endogenous ligands, acting as a sentinel system to “normalise” DA neuron firing, which shifts bidirectionally following cocaine exposure and withdrawal^34,35^. Intriguingly, both partial and full TAAR1 activation exhibit desirable behavioural effects in animal models of cocaine addiction. Our recent observations indicated that both partial and full TAAR1 agonists prevented the decreasing effects of cocaine on brain reward thresholds^11^, produced a downward shift in the dose-response curve for cocaine self-administration^11^ and blocked the spontaneous renewal of cocaine seeking following chronic self-administration^12^. Additionally, neurochemical studies have recently shown that TAAR1 activation reduced DA overflow in the NAc induced by cocaine *in vitro* ^12^ and by methamphetamine both *in vitro* ^36^ and *in vivo* ^37^. Understanding the molecular mechanisms underlying this remarkable ability of TAAR1 to regulate the neurochemical and behavioural effects of psychomotor stimulants is a key step towards the development of more efficacious, TAAR1-based therapies for stimulant addiction.

Notwithstanding the extraordinary diversity of TAAR1 signalling mechanisms and the complexity of TAAR1 molecular interactions, we used fast-scan cyclic voltammetry to measure DA fluctuations induced by cocaine in the NAc, studied the effects of full TAAR1 activation on cocaine’s neurochemical actions and explored the role played by the key TAAR1-associated biochemical cascades. In agreement with previous neurochemical studies^12^, we found here that TAAR1 activation prevented the effects of cocaine on DA transmission, at concentrations of cocaine that produced pronounced effects on DA uptake and no effects on release. When activated by TAs and other endogenous and exogenous ligands, TAAR1 increases cAMP in the presynaptic neuron via Gα_s_-protein activation of adenylyl cyclase which, in turn, promotes PKA and PKC phosphorylation. In heterologous expression systems both PKA and PKC phosphorylation can result in DAT internalization (i.e. noncompetitive reuptake inhibition)^38,39^, thus potentially elevating extracellular DA levels and preventing cocaine binding to the DAT. In addition, PKC activation can induce DAT function to reverse, leading to DA efflux^38^. In fact, TAAR1-dependent DA efflux has been attributed to TAAR1-mediated substrate phosphorylation^40^. These findings suggest that PKA/PKC phosphorylation processes, most likely PKC, could be involved in TAAR1 regulation of cocaine’s effects on DA clearance.

At the doses tested we found that, unexpectedly, inhibition, but not activation, of PKA significantly increased peak DA concentrations, and that both activation and inhibition of this kinase increased DA release in the presence of cocaine. Although many downstream effectors are likely to be involved^41^, PKA stimulation inactivates the small GTPase RhoA, which can prevent DAT internalization^42^ and possibly alter peak DA amplitude changes as well as uptake. PKA may also alter intracellular Ca^2^+ dynamics, increasing intracellular Ca^2^+ release, which may impact on DA release, likely via inhibiting the cAMP/PKA cascade after D_2_ receptor stimulation^43^. Moreover, we found that both activation and inhibition of PKA prevented the ability of cocaine to produce alterations in DA clearance suggesting that basal substrate phosphorylation at the DAT is required for cocaine to exert changes in DA transport and, similarly, that excessive PKA-mediated phosphorylation downregulates DAT function, which is in agreement with previous findings^38^. Importantly, PKA inhibition at a dose that was physiologically active (i.e. increasing DA release by itself) failed to alter the ability of TAAR1 activation to block cocaine’s effects on tau, indicating that TAAR1 modulation of cocaine’s action is unlikely to be mediated by PKA.

Although multiple mechanisms have been linked to PKC-stimulated DAT endocytosis^44^, PKC activation with phorbol esters or G_q_-coupled glutamate receptors stimulated DA efflux in slices of rat striatum^45^. At the doses we tested PKC stimulation did not enhance DA release when applied alone, but did in the presence of cocaine. As with PKA, both PKC activation and inhibition dose-dependently disrupted the ability of cocaine to prolong DA transmission. This suggests that basal substrate phosphorylation is essential but also that PKC-dependent endocytic downregulation^46^, kinetic downregulation of DAT and alterations in DAT conformational equilibrium^47^, which is accompanied by decreased cocaine analogue affinity, may all underlie the reduced ability of cocaine to induce DA transport changes in the presence of PKC activation. Again, inhibition of PKC with Go 6983, at a dose that was neurochemically active (i.e. elevated DA release in the presence of RO5256390) did not prevent TAAR1 activation from blocking cocaine’s effects on DA clearance, suggesting that PKC does not mediate TAAR1’s regulation of cocaine effects.

Strong evidence implicates DA D_2_ autoreceptors in the release and uptake of DA during neurotransmission^48^ and in the neurochemical effects of cocaine. For example, deletion of DA D_2_ autoreceptors amplified the actions of cocaine on DA transmission in the NAc^49^. Our observations were consistent with these findings since the DA D_2_ agonist, sumanirole, decreased the effects of cocaine on DA uptake whereas the antagonist, L-741,626, dose-dependently increased DA release and tau following cocaine treatment. Importantly, L-741,626, at a dose that did not alter cocaine effects on clearance but mildly elevated cocaine effects on release, fully rescued the ability of cocaine to elevate DA transmission in the presence of full TAAR1 activation. These data demonstrate that TAAR1 and DA D_2_ receptors act cooperatively to suppress cocaine’s neurochemical actions. In support of this hypothesis, previous data have shown that in heterologous expressing systems, the presence of DA D_2_ receptors decreased TAAR1-mediated cAMP signalling while enhancing TAAR1-suppressing effects on GSK-3^17^. We therefore predicted that GSK-3 inhibition with SB216763 would reproduce the effects of full TAAR1 activation on cocaine’s actions on DA transmission. This prediction was confirmed fully, thus suggesting that TAAR1/DA D_2_ receptor interactions critically regulate cocaine’s neurochemical actions through silencing of the β-arrestin2-AKT-GSK-3 pathway. This is consistent with an emerging role of GSK-3 in mediating key behavioural actions of cocaine, such as sensitization^50^ and conditioned reward^51^. It must be noted however, that although GSK-3 inhibition did mimic the effects of TAAR1 activation, both when applied alone and/or in combination with cocaine, we acknowledge that GSK-3 is involved in several signalling pathways which could operate independently of TAAR1. Future experiments may thus look at selective GSK-3 activation in this model, which we would predict would prevent the effects of TAAR1 on cocaine as seen here with the D_2_ antagonist. No selective activator of GSK-3 is currently available.

In summary, the present study demonstrated the ability of full TAAR1 activation to modulate DA transmission and prevent cocaine-induced changes on DA neurotransmission. Furthermore, these findings also identified the likely molecular mechanisms through which TAAR1 exerts such remarkable effects, thus uncovering a novel strategy to disrupt cocaine’s actions in the brain. Indeed, TAAR1/DA D_2_ receptor interactions, and their downstream signalling pathways converging on GSK-3, may provide alternative targets for the development of new treatments not only for stimulant addiction but also for other disorders involving DA dysfunction, including schizophrenia and bipolar disorder.
